# Supplementary material for: Predictive role of systemic immune-inflammation index in the prognosis of patients with advanced left-sided colorectal cancer: a retrospective study
Source: PeerJ. 2025 Oct 6;13:e20095. doi: 10.7717/peerj.20095 (PMC12510246; doi:10.7717/peerj.20095)
Supplement: Supplemental Information 5 — † Cutoff values were derived in one subset, applied to the other, and tested with a subset-stratified log-rank test on the merged data; P < 0.05 denotes a significant survival difference. Abbreviations: SII, systemic immune-inflammation index; NLR, neutrophil-to-lymphocyte ratio; PLR, platelet-to-lymphocyte ratio; MLR, monocyte-to-lymphocyte ratio; BMI, body mass index. [file peerj-13-20095-s005.docx]

| Variables | Optimal cutoff value | Stratified log‑rank χ²† | *P*‑value |
| --- | --- | --- | --- |
| SII | 1424.8 | 15.182 | *<0.001* |
| NLR | 2.8 | 7.617 | *0.006* |
| PLR | 184.4 | 7.160 | *0.007* |
| MLR | 0.2 | 14.800 | *<0.001* |
| Age | 67 | 8.660 | *0.003* |
| BMI | 20 | 1.167 | *0.280* |
