# Supplementary material for: Predictive role of systemic immune-inflammation index in the prognosis of patients with advanced left-sided colorectal cancer: a retrospective study
Source: PeerJ. 2025 Oct 6;13:e20095. doi: 10.7717/peerj.20095 (PMC12510246; doi:10.7717/peerj.20095)
Supplement: Supplemental Information 6 — P < 0.05 denotes a significant survival difference. Abbreviations: SII, systemic immune-inflammation index; NLR, neutrophil-to-lymphocyte ratio; PLR, platelet-to-lymphocyte ratio; MLR, monocyte-to-lymphocyte ratio; BMI, body mass index. [file peerj-13-20095-s006.docx]

| Clinicopathological Characteristics | NLR | | *P-value* | PLR | | *P-value* | MLR | | *P-value* |
| --- | --- | --- | --- | --- | --- | --- | --- | --- | --- |
|  | **Low** | **High** |  | **Low** | **High** |  | **Low** | **High** |  |
| Gender |  |  | *0.018^*^* |  |  | *0.164* |  |  | *0.034** |
| Male | 83 | 79 |  | 102 | 60 |  | 45 | 117 |  |
| Female | 47 | 22 |  | 50 | 19 |  | 29 | 40 |  |
| Age (years) |  |  | *0.157* |  |  | *0.271* |  |  | *0.363* |
| ≤67 | 84 | 56 |  | 96 | 44 |  | 48 | 92 |  |
| >67 | 46 | 45 |  | 56 | 35 |  | 26 | 65 |  |
| BMI(Kg/m^2^) |  |  | *0.710* |  |  | *0.084* |  |  | *0.038^*^* |
| ≤23 | 23 | 16 |  | 21 | 18 |  | 18 | 21 |  |
| >23 | 107 | 85 |  | 131 | 61 |  | 56 | 136 |  |
| Treatment Regimen |  |  | *0.024^*^* |  |  | *0.146* |  |  | *0.005^*^* |
| CAPEOX | 63 | 34 |  | 69 | 28 |  | 41 | 56 |  |
| CAPEOX + Bev | 67 | 67 |  | 83 | 51 |  | 33 | 101 |  |
| Primary Tumor Resection |  |  | *<0.001^*^* |  |  | *0.005^*^* |  |  | *0.001^*^* |
| No | 37 | 57 |  | 52 | 42 |  | 19 | 75 |  |
| Yes | 93 | 44 |  | 100 | 37 |  | 55 | 82 |  |
| Site of Metastasis |  |  | *0.245* |  |  | *0.324* |  |  | *0.056* |
| Limited to Liver | 44 | 27 |  | 50 | 21 |  | 29 | 42 |  |
| Not Limited to Liver | 86 | 74 |  | 102 | 58 |  | 45 | 115 |  |
| RAS/BRAF Status |  |  | *0.344* |  |  | *0.206* |  |  | *0.500* |
| Wild-Type | 30 | 21 |  | 38 | 13 |  | 19 | 32 |  |
| Mutant | 56 | 53 |  | 66 | 43 |  | 31 | 78 |  |
| Not Tested | 44 | 27 |  | 48 | 23 |  | 24 | 47 |  |
| Prior Adjuvant Therapy |  |  | *0.504* |  |  | *0.273* |  |  | *0.676* |
| No | 105 | 85 |  | 122 | 68 |  | 62 | 128 |  |
| Yes | 25 | 16 |  | 30 | 11 |  | 12 | 29 |  |
