# Supplementary material for: Predictive role of systemic immune-inflammation index in the prognosis of patients with advanced left-sided colorectal cancer: a retrospective study
Source: PeerJ. 2025 Oct 6;13:e20095. doi: 10.7717/peerj.20095 (PMC12510246; doi:10.7717/peerj.20095)
Supplement: Supplemental Information 7 — * P < 0.05 indicates a significant departure from normality. Abbreviations: NLR, neutrophil-to-lymphocyte ratio; PLR, platelet-to-lymphocyte ratio; MLR, monocyte-to-lymphocyte ratio; SII, systemic immune-inflammation index. [file peerj-13-20095-s007.docx]

| Variable | Shapiro–Wilk *W* | *P*‑value |
| --- | --- | --- |
| NLR | 0.631 | < 0.001^*^ |
| PLR | 0.599 | < 0.001^*^ |
| MLR | 0.588 | < 0.001^*^ |
| SII | 0.350 | < 0.001^*^ |
