# Supplementary figures and images for: Predictive role of systemic immune-inflammation index in the prognosis of patients with advanced left-sided colorectal cancer: a retrospective study
Source: PeerJ. 2025 Oct 6;13:e20095. doi: 10.7717/peerj.20095 (PMC12510246; doi:10.7717/peerj.20095)

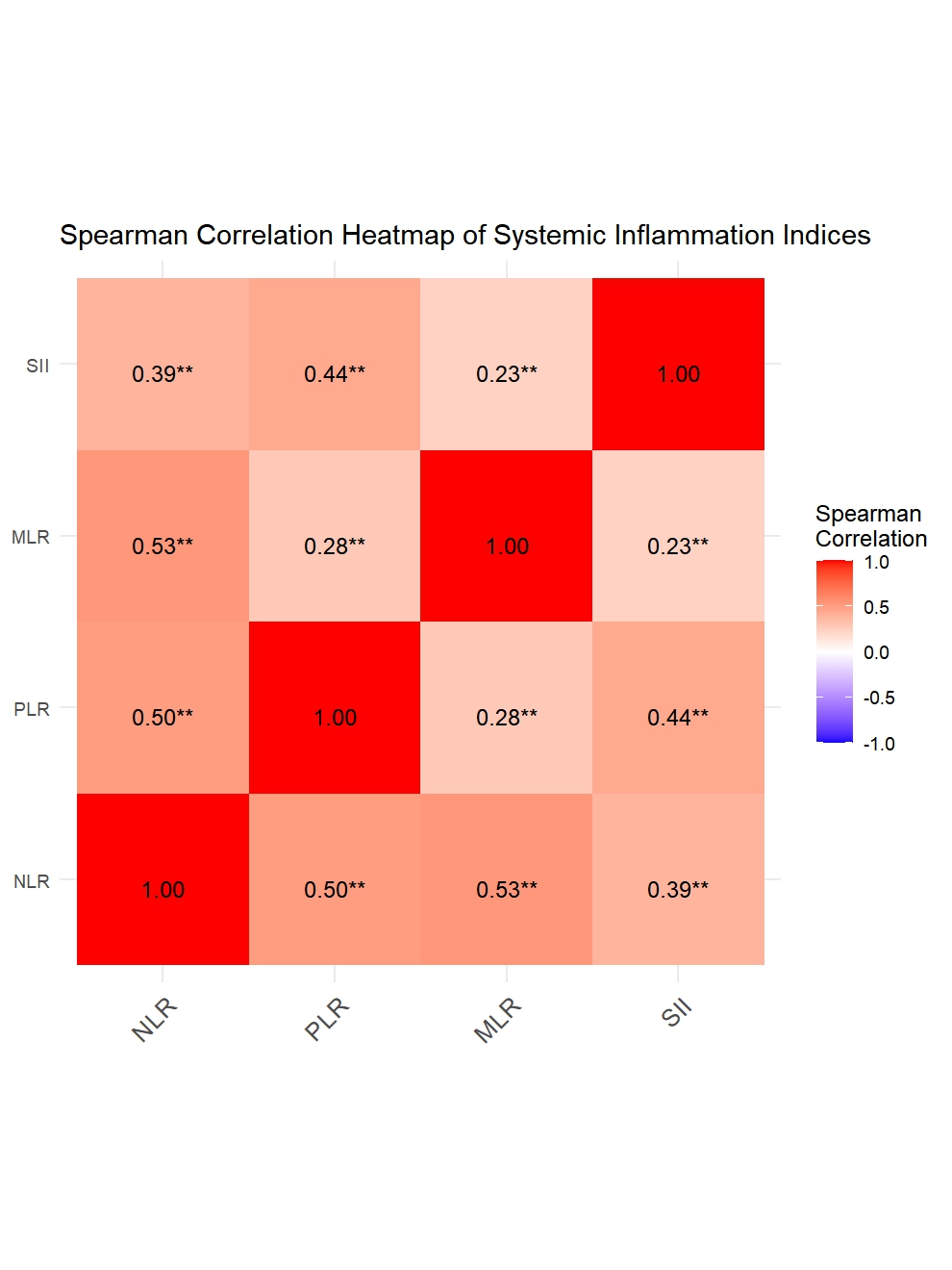

Supplement: Supplemental Information 8 — Each cell shows the Spearman rank-correlation coefficient (ρ) between two indices; deeper red indicates a stronger positive association (scale −1 to +1). Asterisks denote statistical significance (P < 0.01). Abbreviations: NLR, neutrophil-to-lymphocyte ratio; PLR, platelet-to-lymphocyte ratio; MLR, monocyte-to-lymphocyte ratio; SII, systemic immune-inflammation index. [file peerj-13-20095-s008.jpeg]
